# The impact of respiration and oxidative stress response on recombinant α-amylase production by *Saccharomyces cerevisiae*

**DOI:** 10.1016/j.meteno.2016.06.003

**Published:** 2016-06-27

**Authors:** José L. Martínez, Eugenio Meza, Dina Petranovic, Jens Nielsen

**Affiliations:** a Novo Nordisk Foundation Center for Biosustainability, Chalmers University of Technology, SE41296 Gothenburg, Sweden; b Department of Biology and Biological Engineering, Chalmers University of Technology, Kemivägen 10, 41296 Göteborg, Sweden; c Novo Nordisk Foundation Center for Biosustainability, Technical University of Denmark, DK2970 Hørsholm, Denmark

**Keywords:** Yeast, Oxidative stress response, Protein production, Hap1, Amylase

## Abstract

Studying protein production is important for fundamental research on cell biology and applied research for biotechnology. Yeast *Saccharomyces cerevisiae* is an attractive workhorse for production of recombinant proteins as it does not secrete many endogenous proteins and it is therefore easy to purify a secreted product. However, recombinant production at high rates represents a significant metabolic burden for the yeast cells, which results in oxidative stress and ultimately affects the protein production capacity. Here we describe a method to reduce the overall oxidative stress by overexpressing the endogenous *HAP1* gene in a *S. cerevisiae* strain overproducing recombinant α-amylase. We demonstrate how Hap1p can activate a set of oxidative stress response genes and meanwhile contribute to increase the metabolic rate of the yeast strains, therefore mitigating the negative effect of the ROS accumulation associated to protein folding and hence increasing the production capacity during batch fermentations.

## Highlights

• Recombinant protein production is a multi-billion dollar market.
• Heterologous production by yeast generates oxidative stress regardless the target.
• HAP1 overexpression mitigates oxidative stress while enhancing metabolic capacity.
• Overexpression of HAP1 allows higher volumetric productivity of recombinant proteins.
• Strains overexpressing HAP1 may grow in chemostats operated at higher dilution rates.

## 1 Introduction

Proteins are essential cellular components and play many roles in cell physiology, such as scaffolding cell structures, catalysis, signaling, transport and gene expression regulation. Investigating and assessing these roles often requires a deep understanding of protein structure and activity which demands sufficient quantities of the purified protein, which in many cases needs to be produced heterologously (Bill, 2014). Apart from fundamental research, recombinant protein production also represents a multi-billion dollar market (Martinez et al., 2012), due to the use of proteins as biopharmaceuticals and industrial enzymes, e.g. anti-TNF and cancer antibodies, insulin analogs and proteases among others, constituting about 50–60% of the total market (Demain and Vaishnav, 2009, Huang et al., 2014).

*Escherichia coli* is predominantly used as host for commercial production and research (Mattanovich et al., 2012), mainly because it is easy to cultivate and manipulate, due to the large number of molecular tools and genetic information available and, more importantly, due to the fact that the recombinant protein fraction in this organism can reach up to 50% of the dry biomass (Porro et al., 2011). There are, however, important drawbacks of using *E. coli* as a production platform for proteins, for example increased downstream processing costs due to the formation of inclusion bodies (Sorensen, 2010) and lack of correct posttranslational modifications of eukaryal proteins (Ferrer-Miralles et al., 2009). As an alternative, yeasts including *Saccharomyces cerevisiae*, are being increasingly used as the preferred platform for protein production. Yeasts have all the advantages of a microbial system (easy of manipulation and cultivation) and possess eukaryal post-translational machinery (Bill, 2014). Furthermore, the recombinant proteins can be secreted to the extracellular medium, thus facilitating subsequent purification (Nielsen, 2013).

However, one of the limitations of using yeast as a production platform is the burden that secreting proteins at higher rates may represent for cell metabolism, especially for the endoplasmic reticulum (ER), since protein folding is a very crucial step of the secretory pathway: a correct folding determines if the newly synthesized protein is targeted for secretion, otherwise it will be assigned for ER-associated degradation (ERAD) (Schroder, 2008). Thus, larger and more complex proteins produced at high levels often result in induction of ER-stress, and these conditions in the secretory pathway have consequences for the whole cell (Tu and Weissman, 2004). In many cases, protein folding includes disulfide bonds formation, which in eukaryotes is managed by a coordinated action of protein disulfide isomerases (PDI) and Ero1p, using molecular oxygen as the terminal electron acceptor (Tu et al., 2000), hence generating reactive oxygen species (ROS). Often during this process, non-native disulfide bonds are formed, which must be then broken and subsequently re-arranged to form the correct ones, resulting in increased amounts of intracellular ROS. The outcome may be worse if the protein has an odd number of cysteine residues, because it may trigger a futile cycle that leads to oxidative stress (Tyo et al., 2012). Nevertheless, the link between high rates of recombinant protein production and oxidative stress is not just restricted to proteins containing disulfide bonds. Heterologous proteins that are overexpressed need folding assistance even if they do not form disulfide bonds and this can sequester the folding capacity in the ER, creating misfolding of homologous proteins which can limit the efficiency of protein synthesis and result in ROS accumulation (Tyo et al., 2012). Thus, as a rule of thumb, production of secreted heterologous proteins will often generate oxidative stress, regardless of the target protein.

It is generally believed that alleviating ER stress might result in an improved secretion of a desired protein. Thus, several such strategies have been pursued as, for example, overexpression of protein disulfide isomerases (Robinson et al., 1994) or heat shock proteins acting as chaperones for the folding process (Hou et al., 2013, Hou et al., 2014). However, even considering that these strategies increased the secretion efficiency, they did not address in detail the problem of ROS accumulation, which still remains high, and the induction of the oxidative stress response.

In the yeast *S. cerevisiae*, aerobic metabolism is managed by a single transcription factor, encoded by *HAP1*. Hap1p is responsible for the activation of the respiratory metabolism in response to environmental oxygen levels, via heme signaling, as well as the activation of a cluster of oxidative stress responsive genes (Zhang and Hach, 1999), which has been previously reported to be a key player concerning heterologous protein production and regulation of stress levels in yeast systems (Yu et al., 2010, Martinez et al., 2015).

Here we describe a strategy to reduce overall ROS levels while producing proteins at high rates. To achieve this, we chose to overexpress *HAP1* in a *S. cerevisiae* strain overproducing recombinant α-amylase, since this protein has been already successfully used as a model to identify targets for further engineering the secretory pathway and it is known to induce oxidative stress (Tyo et al., 2012, Hou et al., 2014).

## 2 Materials and methods

### 2.1 Yeast strains

For this study, the *S. cerevisiae* strain CENPK.530–1D producing recombinant α-amylase (Liu et al., 2012) was transformed with the plasmid pRS426 (Hou et al., 2012) overexpressing *HAP1*, cloned into the *Bam*HI/*Spe*I sites, downstream the constitutive TEF promoter. The *HAP1* gene sequence was obtained by PCR using *S. cerevisiae* CENPK-7D genomic DNA as template, and the following primers:

5′GGACTAGTCCAGTCGCAGGCAAGAAGGTAAGG3′ (forward primer).

5′CGGGATCCCGTTATTCCAAATTGGAAAATCAGCTCTATAAAAATCAACTAAACCATCAATTTCAACATTATCAACGGGTAG3′ (reverse primer).

The 5′ end of the reverse primer additionally includes a DNA tail (underlined 51 nucleotides) corresponding to the N-terminal of Hap1p inferred from the *S. cerevisiae* W303 strain amino acid sequence, reported as the most efficient version of Hap1 among all the *S. cerevisiae* subtypes (Gaisne et al., 1999). Two clones, namely JLM-1 and JLM-2, were selected for further characterization and analysis.

### 2.2 Physiological characterization in shake flasks and bioreactors

Yeast seed cultures were prepared from fresh individual colonies on agar plates and grown in shake flasks (250 ml) containing liquid YPD supplemented with 2% glucose, overnight, at 30 °C with constant shaking (200 rpm). Batch and chemostat cultures were performed in stirred bioreactors (DASGIP Parallel Bioreactor Systems), yeast pre-cultures inoculated at an initial OD of 0.05 into a final volume of 500 ml of liquid SD ura^−^ his^−^(6.7 g/L YNB without aminoacids +0.75 g/L supplement mix without uracil and histidine, Formedium), containing 2% glucose as carbon source, 2 g/L KH_2_PO_4_, 1 g/L Bovine Serum Albumin (Sigma) and supplemented with 2XSCAA solution (Wittrup and Benig, 1994).

Batch cultures were performed keeping a constant temperature of 30 °C, 600 rpm agitation, standard 1 vvm air flow (21% oxygen blend) and pH controlled at 6 by KOH (2 M). Dissolved oxygen was controlled above 30% using a polarographic oxygen electrode (Mettler Toledo, Switzerland). Real time exhaust oxygen and carbon dioxide values during cultivations were measured by using a DASGIP GA4 analyzer linked to the bioreactor vessels.

Chemostat cultivations were performed at dilution rates of 0.1 and 0.2 h^−1^. The feeding phase (carbon limited, 1% glucose) was initiated right after the remaining ethanol from the batch fermentation was exhausted, and 5 resident times were needed to reach steady state continuous cultivation. All fermentations were done in biological triplicates.

### 2.3 Analytical methods for measuring the exometabolome, secreted amylase and intracellular heme

The dry cell weight was obtained by filtering 5 ml of cell culture through 0.45 µm filters (Sartorius). Throughout the cultivation process, concentrations of glucose, glycerol, ethanol and acetate were analyzed by HPLC (Ultimate 3000, Dionex) equipped with an Aminex HPX-87 h column (Biorad) at 65 °C, using 5 mM H_2_SO_4_ as the mobile phase and a flow rate of 0.6 ml/min.

For the secreted amylase measurements, cells were pelleted by centrifugation from 1 ml of culture broth and the supernatant was taken to determine the amylase concentration by spectrophotometry with the Ceralpha kit (Megazyme), using α-amylase from *Aspergillus oryzae* (Sigma) as standard.

The relative concentration of intracellular heme was determined as described by (Liu et al., 2014). Briefly, an amount of cells corresponding to OD_600_=8 were harvested from the culture, pelleted by centrifugation, resuspended in 500 µl of oxalic acid (20 mM) and stored overnight at 4 °C in an amber tube in dark. Just before the measurement, additional 500 µl of oxalic acid (2 M) was added to each sample, and half of the mixture was taken apart and kept at room temperature whereas the other half was heated up to 95 °C for 30 min. Then 200 µl of clear supernatant was transferred to a 96-well plate to measure the fluorescence (excitation at 400 nm, emission at 600 nm), total heme levels corresponding to the heated samples and free heme to the non-heated samples.

### 2.4 Assessment of gene transcription using real time qPCR

Samples for RNA extraction were taken during exponential phase (batch fermentation) and steady state (chemostat cultivation). Yeast biomass corresponding to 2×10^7^ cells was harvested by centrifugation at 12000x*g* for the RNA extraction using the RNeasy® kit from QIAGEN according to the manufacturer's instructions. A DNAase treatment was performed to remove all traces of remaining genomic DNA (Udvardi et al., 2008, Nolan et al., 2006, Bustin et al., 2009). To assess the concentration and quality of the RNA, the nanodrop 2000 spectrophotometer was used determining the absorbance at 260 nm (nucleic acids), 280 nm (protein) and 230 nm (carbohydrates and other contaminants) (Becker et al., 2010). In order to verify the RNA degradation status in the samples, a 1% agarose gel was used to visualize the 28S, 18S and 5S ribosomal RNA bands. The RNA concentration was set to 500 ng/µl with RNAase-free water and 10 µl were used for first strand cDNA synthesis using the Quantitec kit from QIAGEN following the manufacturer's recommendations. Primers for the test genes were designed using the primer3 software (Rozen and Skaletsky, 2000). The validation of the primers was done with serial 10-fold dilutions of the cDNA and then the qPCR was performed including a melting curve test. The primer dimers existence and the efficiency of the PCR reaction were determined (Ruijter et al., 2009, Schefe et al., 2006). The gene *ARG2* was used as a reference gene. The genes *HAP1*, *CYC1*, *ROX1*, *SOD1* and *YHB1* were used to assess the transcriptional response to *HAP1* overexpression.

The qPCR reactions were performed in the Mx 3005 P thermocycler (Agilent technologies) using Brilliant III Ultra-Fast SYBR® Green QPCR mix, following the manufacturer's instructions. The threshold and the base line were set by default and the C_t_ obtained using the MxPro software (Agilent). The C_t_ values were used to determine the transcriptional fold changes with the ∆C_t_ method (Schefe et al., 2006), using as efficiency value the efficiency obtained from the primer validation.

### 2.5 Assessing cell stress

2×10^7^ cells were harvested from the bioreactor cultures by centrifugation. The cell pellets were resuspended and used for staining with the Mitotracker green FM and the dihydrorhodamine 123 (DHR123) dyes for assessing the mitochondrial homeostasis and the reactive oxygen species accumulation, respectively (Millard et al., 1997, Keij et al., 2000, Qin et al., 2008).

Images from stained cells were taken on an inverted Leica AF 6000 fluorescence microscope with a HCX PL APO CS 100.0×1.40 OIL objective, captured with a DFC 360 FX camera and the Leica Application Suite software. The quantification of the cells involved the analysis of at least 300 cells per sample from three independent experiments. The brightness and the gain settings were adjusted to avoid the background noise and to discard false positives during the counting.

For the determination of the mitochondrial and the cell wall integrity, the cell pellet was suspended in 1 ml of HEPES buffer (HEPES=10 mM, glucose 5%, pH=7.4) containing the dye mitotracker green FM (100 nM) (Keij et al., 2000). The cell suspension was incubated at room temperature in dark for 15 min. After the incubation, the cells were centrifuged at 12000x*g* and 2 µl of the cell pellet was loaded on a microscope slide for inspection by fluorescence microscopy using the filters for green and red fluorescence.

The reactive oxygen species (ROS) accumulation was determined by staining the cells with DHR123 (Qin et al., 2008). The cell pellet was washed three times with water and suspended in 1 ml of sodium citrate buffer (sodium citrate 50 mM, glucose 2%, pH=5.0) containing DHR123 (50 µM). The cell suspension was incubated at room temperature in dark for 15 min. After the incubation, the cells were centrifuged at 12000x*g* and 2 µl of the cell pellet was loaded on a microscope slide for inspection by fluorescence microscopy using the filter for yellow fluorescence.

### 2.6 Cell size determination

To determine the population cell size, the Guava easyCyte™ HT System was used. The forward scatter (FSC) and the side scatter (SSC) were used as parameters for cell size and shape respectively. The control was the strain with no overexpression of the gene *HAP1*, whereas the two strains harboring the plasmid that overexpress *HAP1* were treated as the experiments (referred as JLM-1 and JLM-2). Prior to the analysis, the cytometer performance was assessed using the guava easyCheck™ Kit. The FCS 3.0 file was converted to a FCS 2.0 file using the FCS convert utility from http://www.guavatechsupport.com/muse/. Once converted, the FCS 2.0 file was analyzed and plotted using the flowCore and the ggplot software (Ellis pH et al.,, Wickham, 2009).

## 3 Results and discussion

### 3.1 *HAP1* overexpression promotes respiration in a dose-dependent manner

**Fig. 1:**
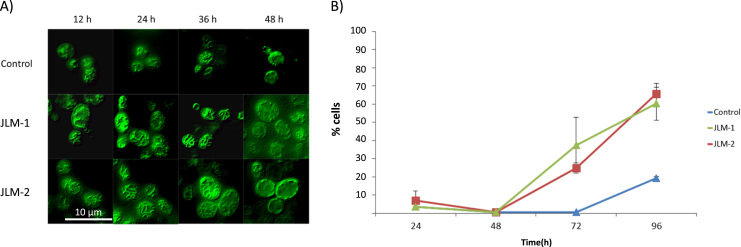
(A) Mitochondrial network display exhibited by control and *HAP1* overexpressing cells (JLM-1 and JLM-2) throughout the fermentation process as observed by fluorescence microscopy. A 10 µm scale bar is displayed for cell size reference. (B) ROS accumulation estimated (percentage of cells affected over the total cell population) for the studied strains at different sample points during the fermentation.

Batch fermentations of *S. cerevisiae* strains overexpressing the transcription factor Hap1p (JLM-1 and JLM-2), and the control strain (empty plasmid), were performed in synthetic liquid media using 2% glucose as carbon source. Samples were taken to estimate the physiological parameters at different time points (Table 1), and we found that the *HAP1* overexpressing strains had a higher growth rate and a shorter glucose consumption phase. We also found slight differences between the two Hap1 overexpressing clones: the clone JLM-2 had and even higher growth rate and shorter glucose phase than the clone JLM-1 (Supplementary figure S1). We performed RT-qPCR analysis and found that the two clones had different expression levels of *HAP1* (1.4 fold and 2.1 fold over the control strain for JLM-1 and JLM-2 respectively), probably due to differences in the plasmid copy number, since it is a high copy plasmid and its number may differ among transformant strains up to 5% (www.adgene.org). The strain with the higher expression level of *HAP1* (JLM-2) had a higher growth rate and faster consumption both of glucose and of ethanol. Additionally, *HAP1* overexpressing strains had a lower ethanol yield (higher the expression, lower the yield). This might indicate that increased Hap1p levels may promote diminishment of the fermentative metabolism (preferred by *S. cerevisiae*), and increased respirative metabolism. We assessed the status of the mitochondrial network (Fig. 1A) and found that in *HAP1* overexpressing strains, the mitochondrial network is larger and more developed compared with the control strain, which is in accordance with a higher respiratory rate. We also noticed that the size of the *HAP1* overexpressing cells is slightly bigger, which was further confirmed by flow cytometry (Supplementary Fig. S2).

**Table 1:** Physiological characterization of the α-amylase producing strains during batch fermentation in bioreactors.

Strains	µ_max_^a^ (h^−1^)	Y_SE_^b^	Y_SG_^b^	Y_SA_^b^
Control	0.31	0.32	0.09	0.01
JLM-1	0.35	0.27	0.1	0.01
JLM-2	0.4	0.21	0.07	0.02

Average values from three independent biological replicates for each strain are shown. The calculated standard error calculated for all the cases was less than 3%.

a Maximum specific growth rate (h^−1^) on glucose.

b Yield of ethanol (E), glycerol (G) and acetate (A) from glucose (S) (g/g).

### 3.2 Hap1 promotes increased growth rates without oxidative stress penalty during batch fermentations

Overexpression of the *HAP1* gene increased the metabolic rate of *S. cerevisiae*. Thus, in order to study the consequences of increased respiration on oxidative stress, we determined the production of ROS and mitochondrial damage throughout the fermentation process in bioreactors: yeast cells were grown in batch cultivations and samples were collected at different time points. Results shown in Fig. 1B revealed that the levels of oxidative stress in the *HAP1* overexpressing strains, evidenced by the production of ROS and the mitochondrial dysfunction, were kept at the same level as the control strain during the first 48 h of cultivation. It is after this time, when a clear divergence among the strains analyzed occurs: the transformant cells exhibit an important ROS accumulation and severe mitochondrial damage after 72 h of batch fermentation, which is even more acute after 96 h.

**Fig. 2:**
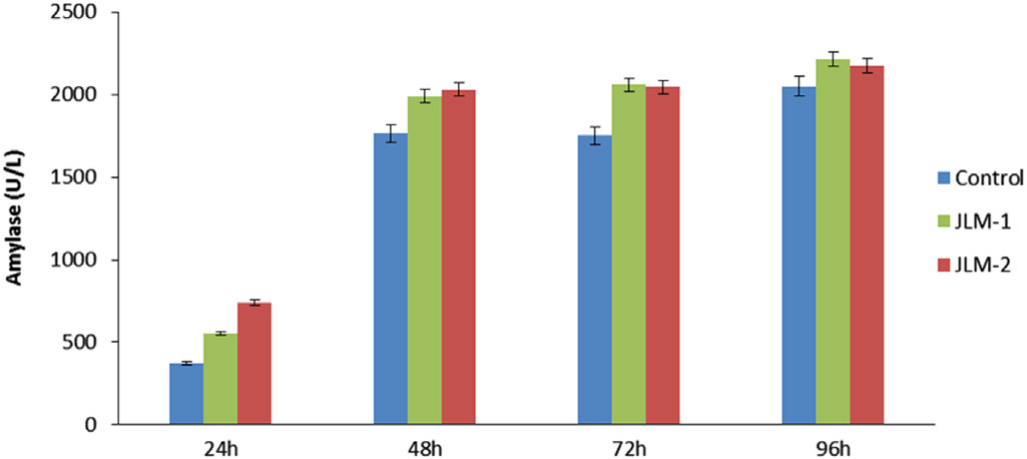
Amylase titer calculated for the *HAP1* overexpressing strains (JLM-1 and JLM-2) compared to the control strain during batch fermentation in the bioreactors.

The amount of recombinant amylase secreted during batch by the *HAP1* overexpressing strains compared to the control strains was also analyzed. Interestingly, the amount of recombinant protein produced by the strains JLM-1 and JLM-2, on average, almost doubles the amount of amylase produced by the control strain: 646 U/L (average) vs. 371 U/L, respectively (Fig. 2). This increase is, however, primarily due to the faster growth of the engineered strain as the specific titer was about the same for the two strains (563 U/g CDW and 561 U/g CDW for the control strain and the JML-1 strain, respectively). The difference in the production capacity among strains still remains significant after 48 h and persists over time throughout the whole fermentation process, but with a smaller difference at the end of the fermentation.

*HAP1* overexpression could increase the growth rate by 20–25% (Table 1) with no additional burden in ROS during the first 48 h of fermentation, as previously shown in Fig. 1B, allowing for higher volumetric productivity of amylase. The transformant strains do not suffer from oxidative stress during this period and they are therefore able to keep a higher rate of protein production, while the control strains show a slower metabolic rate, hence a lower growth rate and therefore a lower protein yield. In summary, the *HAP1* strains produce more protein over time because they can grow faster than the control and for 48 h are unaffected by oxidative stress, just before the ROS levels become unsustainable so they start suffering from mitochondrial damage.

### 3.3 *HAP1* overexpression enhances amylase productivity in chemostat cultivations at increased dilution rates

**Fig. 3:**
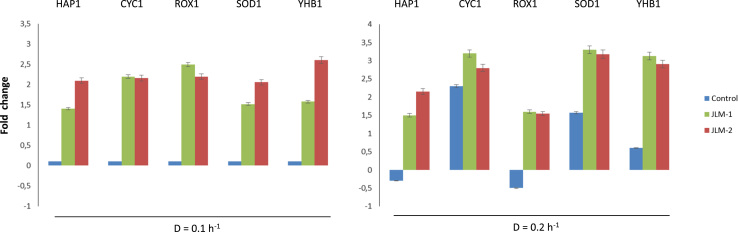
Relative expression levels of *HAP1* of the transformant strains (JLM-1 and JLM-2) and control strains and some of its target genes involved in respirative metabolism (*CYC1* and *ROX1*) and oxidative stress protection (*SOD1* and *YHB1*), estimated by qPCR from samples taken during chemostat cultivation in bioreactors at two different dilution rates (0.1 and 0.2 h^-1^). All the values are relative and normalized to those obtained for the control strain at D=0.1.

In order to assess the oxidative stress response triggered by Hap1p, the relative expression of a few representative genes that are induced by this transcription factor in the oxidative stress response, such as *SOD1* and *YHB1* (Zhang and Hach, 1999, Ter Linde and Steensma, 2002), were analyzed by qPCR. The analysis also included other genes related to the activation of the respiratory metabolism (*CYC1* and *ROX1*). Samples for this analysis were taken from chemostat cultures at two different dilution rates (0.1 h^−1^ and 0.2 h^−1^), after five residence times (time needed for yeast cultures to reach the steady state). In addition, the amylase concentration was measured and the productivity was calculated for each strain at the given dilution rate. As shown in Fig. 3, genes related to oxidative stress protection were overexpressed in JLM-1 and JLM-2 at both dilution rates compared to the control strain, as well as those involved in the respiratory metabolism.

**Fig. 4:**
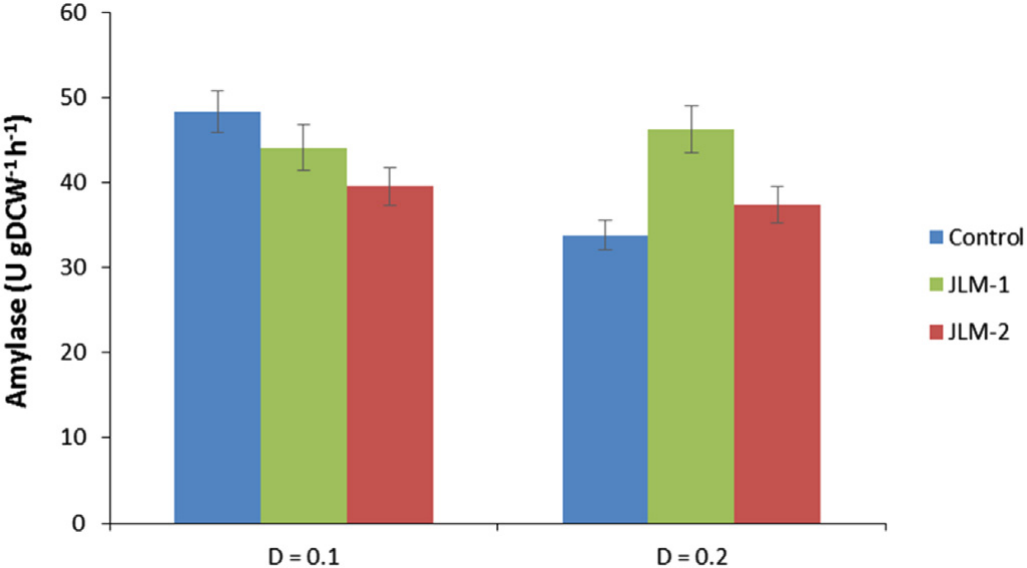
Amylase specific productivity for JLM-1 and JLM-2 compared to the control strain during chemostat cultivations at different dilution rates.

When we measured the amylase productivity, we found that at the lower dilution rate, the control strain shows a higher value than the *HAP1* overexpressing strains (Fig. 4). However, increasing the dilution rate results in a significant decrease of the production capacity for the control strain, whereas the capacity of the strains JLM-1 and JLM-2 remains unaffected, if not slightly improved for the case of JLM-1. From these results we might conclude that, since both transformant strains have an increased growth rate, increasing the dilution rate for the chemostat cultivation has no negative effect because they are still far from their optimal growth capacity (which is µ=0.4 for the best strain), whereas for the case of the control strain, getting close to its maximum growth rate means an increase in the metabolic rate and hence an increased generation of ROS and oxidative stress, which ultimately affects the amylase productivity of this strain. This negative effect on the amylase productivity at increasing dilution rates during chemostat cultivations was already reported by Liu et al. (2013), and the transcriptional analysis suggested that might be related to ER processing and global stress responses. Here we propose that oxidative stress is a significant contributor to this effect in aerobic culture conditions, since higher expression levels of the relevant protective genes controlled by Hap1p may keep the amylase productivity levels intact.

In summary, the *HAP1* overexpressing strains produce more protein over time because they can grow faster than the control, respire more efficiently, and are less affected by oxidative stress and mitochondrial damage due to the continuous induction of the oxidative stress response.

## 4 Conclusion

Regardless the type of protein being over-expressed, high rates of protein production represent a metabolic burden for yeast cells, which implicates several kinds of cell stress, including oxidative stress which results in ROS generation. In order to reduce the negative effect of oxidative stress we generated two different strains over-expressing the transcription factor Hap1p, which activates several oxidative stress response genes. This induced the oxidative stress response and at the same time increased the metabolic capacity (growth rate and respiration) of *S. cerevisiae*. Moreover, the strategy of increasing oxidative stress response by over-expression of *HAP1* allows increased productivity both in batch cultures and in chemostats, which can be operated at a higher dilution rate. The results of our work therefore confirm that there is a direct link between oxidative stress and the recombinant protein production capacity in *S. cerevisiae*, and that the negative effects caused by the increased metabolic burden triggered by protein over-expression can be significantly mitigated simply by the enhancement of the molecular mechanisms already present in the cell.
