# Supplementary material for: The impact of respiration and oxidative stress response on recombinant α-amylase production by Saccharomyces cerevisiae
Source: Metab Eng Commun. 2016 Jun 27;3:205–10. doi: 10.1016/j.meteno.2016.06.003 (PMC5779723; doi:10.1016/j.meteno.2016.06.003)
Supplement: Supplementary file 1 — Supplementary material [file mmc1.pptx]

## Slide 1
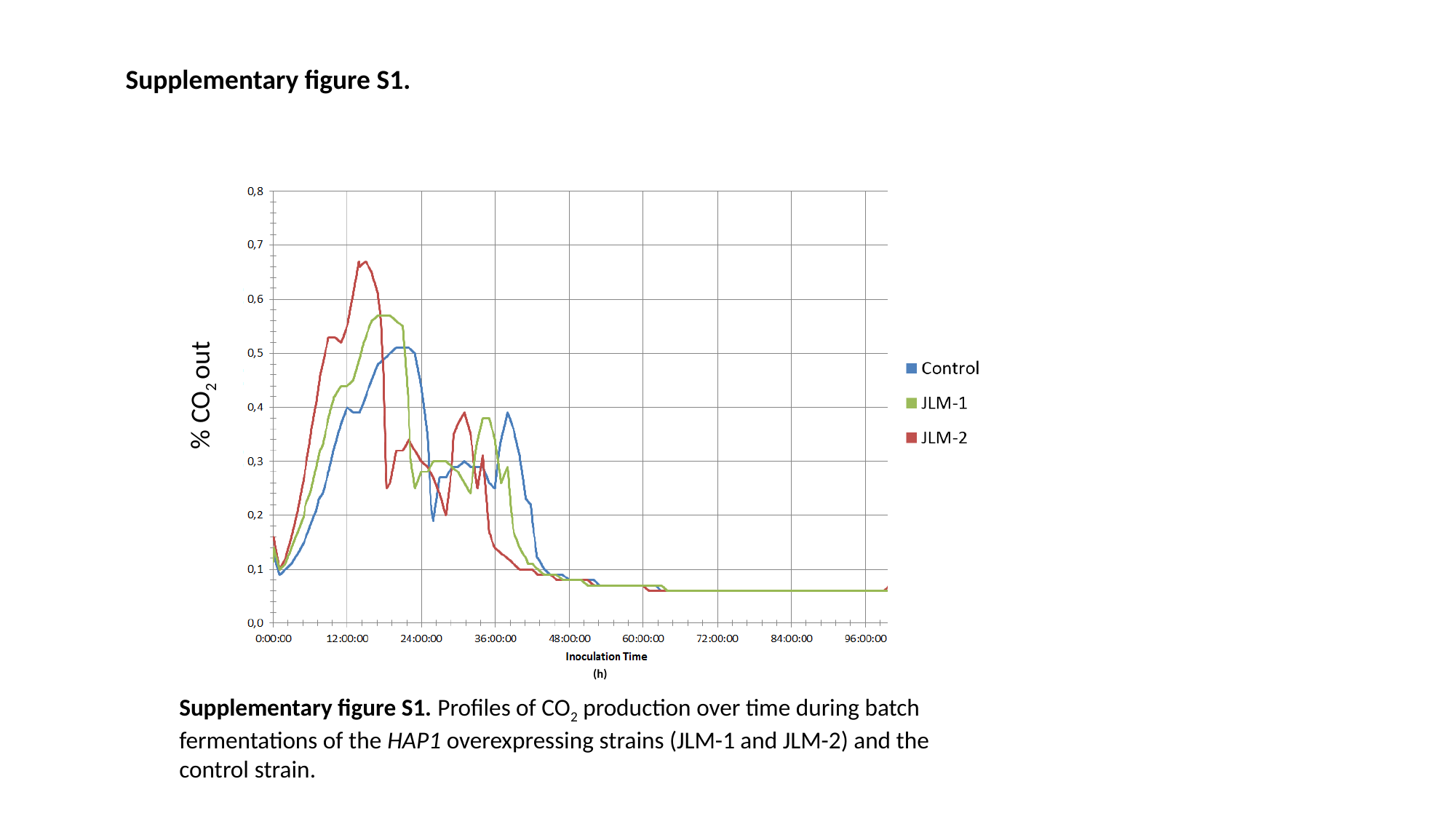

Supplementary figure S1.
% CO2 out
(h)
Supplementary figure S1. Profiles of CO2 production over time during batch fermentations of the HAP1 overexpressing strains (JLM-1 and JLM-2) and the control strain.

## Slide 2
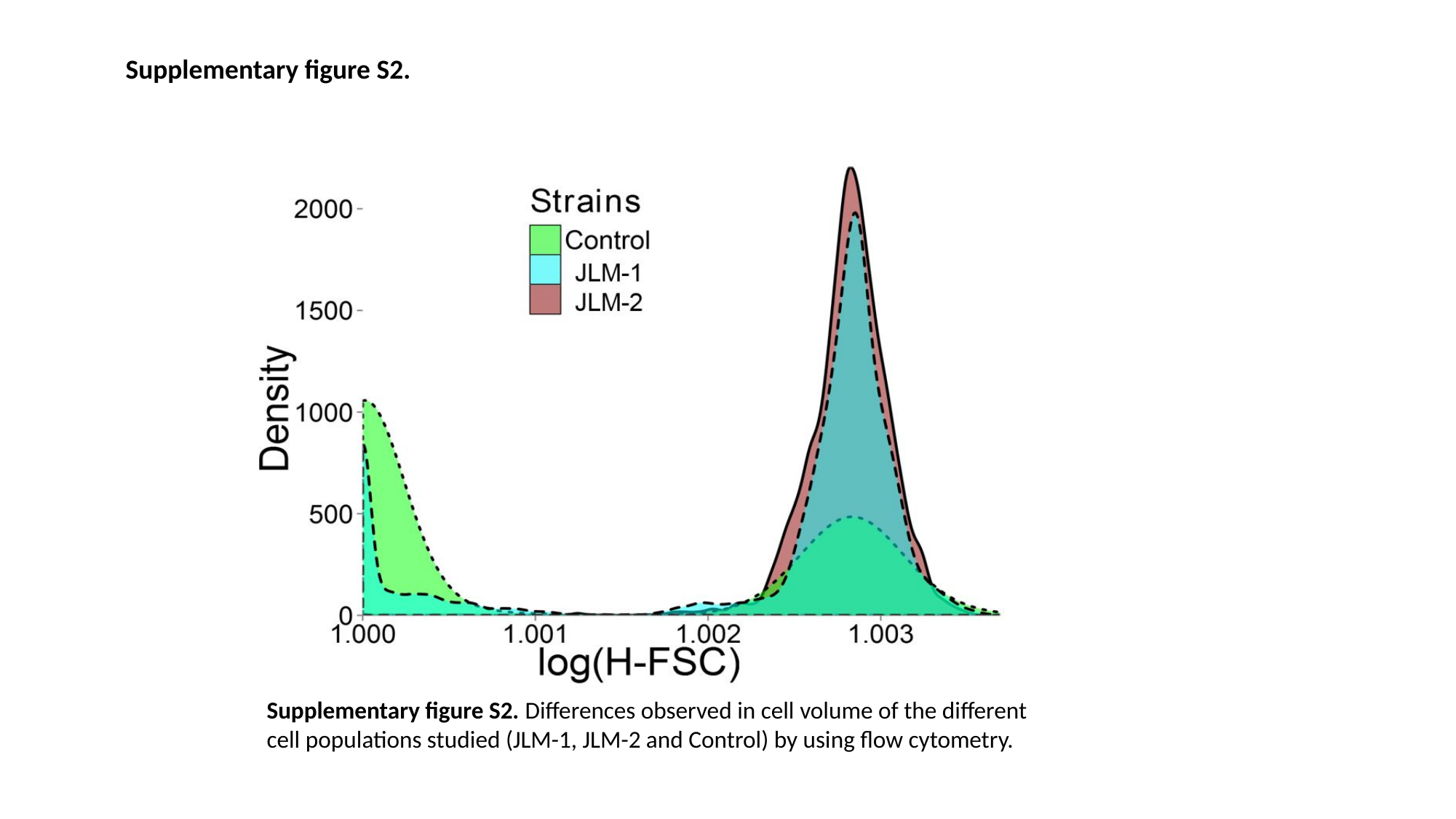

Supplementary figure S2.
Supplementary figure S2. Differences observed in cell volume of the different cell populations studied (JLM-1, JLM-2 and Control) by using flow cytometry.
